# Neuroprotective Effects and Treatment Potential of Incretin Mimetics in a Murine Model of Mild Traumatic Brain Injury

**DOI:** 10.3389/fcell.2019.00356

**Published:** 2020-01-10

**Authors:** Miaad Bader, Yazhou Li, David Tweedie, Nathan A. Shlobin, Adi Bernstein, Vardit Rubovitch, Luis B. Tovar-y-Romo, Richard D. DiMarchi, Barry J. Hoffer, Nigel H. Greig, Chaim G. Pick

**Affiliations:** ^1^Department of Anatomy and Anthropology, Sackler Faculty of Medicine, Tel Aviv University, Tel Aviv, Israel; ^2^Translational Gerontology Branch, Intramural Research Program, National Institute on Aging, National Institutes of Health, Baltimore, MD, United States; ^3^Feinberg School of Medicine, Northwestern University, Chicago, IL, United States; ^4^Division of Neuroscience, Institute of Cellular Physiology, Universidad Nacional Autónoma de México, Mexico City, Mexico; ^5^Department of Chemistry, Indiana University, Bloomington, IN, United States; ^6^Department of Neurosurgery, Case Western Reserve University School of Medicine, Cleveland, OH, United States; ^7^Sagol School of Neuroscience, Tel Aviv University, Tel Aviv, Israel; ^8^Center for the Biology of Addictive Diseases, Tel Aviv University, Tel Aviv, Israel

**Keywords:** glucagon-like peptide-1, glucose-dependent insulinotropic polypeptide, incretin, traumatic brain injury, concussive head injury, twincretin, liraglutide

## Abstract

Traumatic brain injury (TBI) is a commonly occurring injury in sports, victims of motor vehicle accidents, and falls. TBI has become a pressing public health concern with no specific therapeutic treatment. Mild TBI (mTBI), which accounts for approximately 90% of all TBI cases, may frequently lead to long-lasting cognitive, behavioral, and emotional impairments. The incretins glucagon-like peptide-1 (GLP-1) and glucose-dependent insulinotropic polypeptide (GIP) are gastrointestinal hormones that induce glucose-dependent insulin secretion, promote β-cell proliferation, and enhance resistance to apoptosis. GLP-1 mimetics are marketed as treatments for type 2 diabetes mellitus (T2DM) and are well tolerated. Both GLP-1 and GIP mimetics have shown neuroprotective properties in animal models of Parkinson’s and Alzheimer’s disease. The aim of this study is to evaluate the potential neuroprotective effects of liraglutide, a GLP-1 analog, and twincretin, a dual GLP-1R/GIPR agonist, in a murine mTBI model. First, we subjected mice to mTBI using a weight-drop device and, thereafter, administered liraglutide or twincretin as a 7-day regimen of subcutaneous (s.c.) injections. We then investigated the effects of these drugs on mTBI-induced cognitive impairments, neurodegeneration, and neuroinflammation. Finally, we assessed their effects on neuroprotective proteins expression that are downstream to GLP-1R/GIPR activation; specifically, PI3K and PKA phosphorylation. Both drugs ameliorated mTBI-induced cognitive impairments evaluated by the novel object recognition (NOR) and the Y-maze paradigms in which neither anxiety nor locomotor activity were confounds, as the latter were unaffected by either mTBI or drugs. Additionally, both drugs significantly mitigated mTBI-induced neurodegeneration and neuroinflammation, as quantified by immunohistochemical staining with Fluoro-Jade/anti-NeuN and anti-Iba-1 antibodies, respectively. mTBI challenge significantly decreased PKA phosphorylation levels in ipsilateral cortex, which was mitigated by both drugs. However, PI3K phosphorylation was not affected by mTBI. These findings offer a new potential therapeutic approach to treat mTBI, and support further investigation of the neuroprotective effects and mechanism of action of incretin-based therapies for neurological disorders.

## Highlights

- Liraglutide and twincretin ameliorate mTBI-induced cognitive deficits.

- Liraglutide and twincretin mitigate mTBI-induced neurodegeneration.

- Liraglutide and twincretin alleviate mTBI-induced elevation in microglial expression.

- mTBI challenge result in a decline in p-PKA levels in ipsilateral cortex.

- Liraglutide and twincretin attenuate mTBI-induced reduction in p-PKA expression.

## Introduction

Traumatic brain injury (TBI) is a profound public health concern, affecting at least 1.7 million people in the United States alone each year and leading to diverse impairments, hospitalizations, and deaths (Faul et al., 2010). TBI has hence become a major economic burden, as the total annual cost of TBI in the United States including direct costs of medical treatments and deaths, as well as reduced productivity, was estimated to be 60.43 billion U.S. dollars, based on data from 2000 (Finkelstein et al., 2006). The major causes of TBI are falls, vehicle accidents, assaults, and sport injuries (Bruns and Hauser, 2003). These injuries are more frequent among men, especially adolescents and young adults, due to vehicle accidents and alcohol-related trauma, and the elderly due to a higher risk for falls (Moppett, 2007). Diagnosis of TBI relies mainly on clinical symptoms, including level of consciousness and amnesia, as well as CT findings evaluating morphological brain damage following exposure to injury (Tagliaferri et al., 2006; Maas et al., 2008). There is a growing body of evidence suggesting that TBI is a significant risk factor for the development of neurodegenerative diseases such as Parkinson’s and Alzheimer’s disease (Fleminger et al., 2003; Tweedie et al., 2013; Gardner and Yaffe, 2015).

Traumatic brain injury results in a myriad of pathophysiological changes that develop in two phases. Primary brain injury, which is a direct consequence of the external force exerted on the brain, consists of tissue distortion and destruction proximal to the injury and disruption of axons and small vessels, causing immediate necrotic neuronal cell death (Greve and Zink, 2009). Processes initiated during the primary phase lead to progressive and extended secondary damage, including neuroinflammation, oxidative stress, and glutamate excitotoxicity, which all contribute to neuronal apoptotic cell death (Werner and Engelhard, 2007; Greve and Zink, 2009).

Mild TBI (mTBI) accounts for approximately 80–90% of all TBI cases (Levin and Diaz-Arrastia, 2015). Although routine diagnostic evaluations of mTBI patients often fail to show clear structural brain damage, these patients frequently suffer short- and long-lasting cognitive, behavioral, and emotional impairments (Daneshvar et al., 2011). Such impairments include, among others, memory and concentration deficits, poor executive functions, depression, and anxiety-related disorders (Levin and Diaz-Arrastia, 2015). Continuation of these symptoms for 1–3 months post injury has been defined as “post-concussive syndrome” (PCS), whereas continuation for more than 3 months is defined as persistent PCS (PPCS). Some 50% of mTBI casualties suffer from PCS and 15% suffer PPCS for more than 1 year following the injury (Coronado et al., 2012); with a recent report suggesting yet higher rates (McInnes et al., 2017). Whereas clinical mTBI management generally includes assessment, observation, symptomatic treatment, and post-discharge follow-up, there remains a significant need to develop evidence-based interventions to mitigate long-term impairments and disabilities (Levin and Diaz-Arrastia, 2015).

The incretins, glucagon-like peptide-1 (GLP-1) and glucose-dependent insulinotropic polypeptide (GIP), are glucose-lowering, intestinal-derived peptides. Their receptors, GLP-1R and GIPR, are primarily localized to pancreatic islet cells and implicated in the treatment of type 2 diabetes mellitus (T2DM) (Lovshin and Drucker, 2009; Seino et al., 2010). However, both receptors are also present elsewhere and particularly throughout the nervous system, on the dendritic branches of neurons as well as on activated microglia and astrocytic cells (Hamilton and Holscher, 2009; Figueiredo et al., 2011). As incretins act protectively and trophically on β-cells (Trümper et al., 2001; Drucker, 2003; Li et al., 2003) and their receptors are coupled to the cAMP second messenger pathway whose upregulation is associated with neuroprotection and anti-inflammation (Tabuchi et al., 2002; Perry and Greig, 2003; Cui and So, 2004), the potential therapeutic benefit of incretin mimetics in brain pathologies is of growing interest. Our hypothesis is that incretin mimetics provide neurotrophic, protective, and anti-inflammatory processes, similar to their actions in pancreas (Perry et al., 2002; Nyberg et al., 2005; Li et al., 2010b; Paratore et al., 2011).

In animal models, incretin analogs have been efficacious in preclinical models of: (i) Alzheimer’s disease (Li et al., 2010a; McClean et al., 2011; Faivre and Holscher, 2013), (ii) Parkinson’s disease (Bertilsson et al., 2008; Li et al., 2009, 2016), (iii) stroke and ischemia (Li et al., 2009; Hyun Lee et al., 2011), (iv) peripheral neuropathy (Perry et al., 2007), (v) amyotrophic lateral sclerosis (Li et al., 2012), (vi) Huntington’s disease (Martin et al., 2009), and (vii) TBI (Rachmany et al., 2013; Li et al., 2015; Yu et al., 2016; Tamargo et al., 2017; Bader et al., 2019). Moreover, as marketed GLP-1 analogs to treat T2DM are well-tolerated and rarely cause hypoglycemia, clinical trials have been undertaken to investigate the therapeutic effects of exendin-4 or liraglutide on Alzheimer’s disease (Egefjord et al., 2012; Gejl et al., 2016), and Parkinson’s disease patients (Aviles-Olmos et al., 2013; Athauda et al., 2017), and continue to be evaluated.

Previous studies by our group characterized select neuroprotective actions of liraglutide, a GLP-1 analog, and twincretin, a dual GLP-1 and GIP analog. Both analogs augmented cell viability following exposure to oxidative stress and glutamate excitotoxicity in neuronal cell cultures. The cAMP/PKA/CREB pathway apparently played an important role in these neuroprotective actions. Moreover, both agents mitigated short-term cognitive impairments in a concussive mTBI rodent model (Li et al., 2015; Tamargo et al., 2017), indicating translational relevance from cellular studies to *in vivo* ones.

The present study sought to provide a more comprehensive assessment of the potential therapeutic benefits of these incretin-based therapies on the cognitive deficits and brain pathology that occur following mTBI using the same rodent model that mimics key post concussive symptoms in humans. Specifically, the aim of the present study was to evaluate whether liraglutide and twincretin administration of a clinically translatable dose could mitigate mTBI-induced cognitive deficits, neuronal degeneration, and neuroinflammation, and to appraise which downstream cascade of GLP-1R/GIPR activation was primarily responsible for these neuroprotective actions. A secondary aim was to compare these two incretin-related therapeutic approaches to determine whether a therapeutic advantage exists in activating both GLP-1R and GIPR by twincretin in comparison to only the GLP-1R by liraglutide with clinically relevant doses.

## Materials and Methods

### Experimental Animals

Adult ICR mice (6–8 weeks), weighting 31–34 g, were kept five per cage with food and water *ad libitum*, at a constant temperature of 22 ± 5°C and under 12 h light/dark cycle. All experimental procedures were conducted during the light phase of the cycle. Each mouse was used in only one experiment and all efforts were made to minimize potential suffering. The Ethics Committee of the Sackler Faculty of Medicine approved the experimental protocol (M-14-050 and M-15-011), in compliance with the guidelines for animal experimentation of the National Institutes of Health (DHEW publication 85-23, revised, 1995).

### Mild Traumatic Brain Injury Model

Head injury was induced using a weight drop head trauma device, as previously described (Zohar et al., 2003; Milman et al., 2005). This device is composed of a metal tube (80 cm long, 13 cm inner diameter) and a sponge under the tube to support the head of the mice. The mice were lightly anesthetized by inhalation of isoflurane and put under the device. A metal weight (30 g) was then dropped from the top of the 80 cm tube to strike the head of the mouse on the right temporal side between the corner of the eye and the ear. Immediately following the injury, mice were put back in their original cages for recovery. Sham mice were treated similarly to mTBI challenged mice. They were anesthetized by isoflurane and aligned under the weight drop device, but not exposed to the injury. They were then returned to their cages for recovery. Sham and mTBI mice were indistinguishable following their recovery from the procedure. Body temperature has been shown to be maintained in our previous studies with this procedure, and the selection of animal number for individual studies (whether behavioral, immunohistochemical, or biochemical) was determined from the variance in the data of our prior studies.

### Drug Administration

246.7 μg/kg of liraglutide or 50 μg/kg twincretin prepared in isotonic saline was administered to mice once daily in a 7-day regimen of subcutaneous (s.c.) injections, with the first injection given 30 min following the injury. Liraglutide dose (246.7 μg/kg/day) is equivalent to the common human dose (20 μg/kg/day) normalized to body surface area across species based on FDA guidelines (U.S. Department of Health and Human Services et al., 2005). Twincretin dose (50 μg/kg/day) was chosen based on previous studies in mice diabetes model (Finan et al., 2013) and on the manufacturer’s recommendation. Notably, twincretin and liraglutide are used in humans at the same dose; specifically, both at 1.8 mg daily by s.c. administration. Translation of this 1.8 mg dose in an 88.8 kg human across species is approximately 246.7 μg/kg in a mouse for each agent. However, in the current mouse study, the twincretin selected dose was fivefold lower; specifically 50 μg/kg. This dose was based on preliminary studies indicating that it was on the linear portion of the dose–response curve. Mice were randomly divided into four groups: a sham group, an mTBI group, a liraglutide following mTBI group, and a twincretin following mTBI group. Sham and mTBI mice were injected by saline in the same regimen as treatment groups.

### Experimental Procedures

**FIGURE 1:**
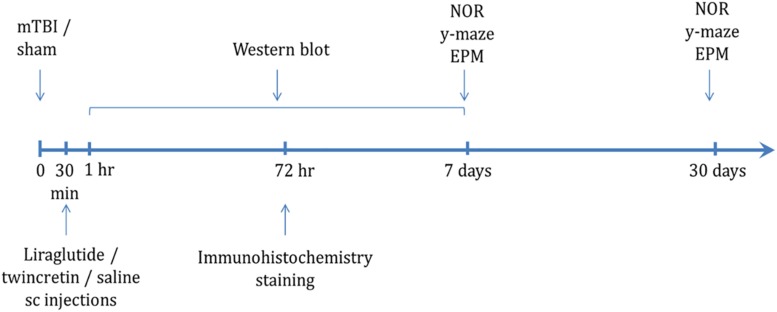
Scheme of study design time line. Animals were exposed to sham/mTBI and 30 min later were given liraglutide (247.6 μg/kg), twincretin (50 μg/kg), or saline once daily via s.c. injections for 7 days {the human dose of liraglutide and twincretin is 1.8 mg s.c. daily that, for a 88.8 kg human [the mean weight of a male American (Yuan et al., 2017)], following normalization to body surface area across species in line with U.S. Department of Health and Human Services Food and Drug Administration guidelines (Han et al., 2016), translates to a mouse dose of approximately 247.6 μg/kg}. Behavioral tests to assess behavior and cognitive abilities were carried out 7 and 30 days following mTBI in separate cohorts of animals. Immunohistochemical staining to evaluate neurodegeneration and neuroinflammation was performed 72 h following mTBI challenge. Western blot analysis to assess changes in neuroprotective protein levels following mTBI and drug treatments was performed starting from 1 h to 1 week post mTBI. Each experimental time point was performed using a different group of mice (NOR: novel object recognition paradigm; EPM: elevated plus maze paradigm).

The timeline of the experimental procedures following exposure to mTBI is shown in Figure 1. Mice were subjected to mTBI and 30 min later, a 7-day regimen of either liraglutide or twincretin treatment was initiated. The behavioral tests were performed at 7 or 30 days following the injury in separate groups of animals. Immunohistochemical staining assessments were performed on brains that were collected 72 h post injury. Western blot analysis was carried out on brain tissue collected from 1 h to 1 week post mTBI.

### Behavioral Tests

Behavioral assessments were conducted 7 or 30 days post mTBI. Each mouse was used for one time point only. Mouse behavior and cognition were assessed using the elevated plus maze, novel object recognition (NOR) and Y-maze paradigms. All equipment used for the behavioral tests was cleaned with 70% ethanol between sessions to minimize any olfactory influences on behavior.

#### Elevated Plus Maze Paradigm

The elevated plus maze was used to evaluate anxiety-like behavior of the mice, as previously described (Zohar et al., 2011). This test relies on the preference of rodents to explore enclosed darkened environments rather than open, bright, and elevated environments. In addition, this test was used to evaluate locomotor activity of the mice. The apparatus consisted of a black, four-armed Pexiglass maze, in which two of the arms have low walls (open—30 × 5 × 1 cm) and the other two arms have high walls (closed—30 × 5 × 15 cm). Similar arms face each other, and all have no roof. Each mouse was put in the middle of the apparatus, facing one of the open arms, and was allowed to explore the arena for 5 min. The amount of time the mice spent in the open arms and the number of entries to the open/closed arms was recorded. Longer duration of time spent within the open arms has been associated with lower anxiety levels (Belzung and Griebel, 2001), while higher number of entries to the open/closed arms is indicative to higher locomotor activity.

#### Novel Object Recognition Paradigm

The NOR task was chosen to assess the recognition and visual memory of the mice, as previously described (Edut et al., 2011). This paradigm relies on the tendency of rodents to investigate novel objects within their environment rather than known ones. This task is used to evaluate whether a mouse is able to discriminate between a familiar and a novel object. The arena consists of an open field black Plexiglass box (59 × 59 × 20 cm size); 48 h prior to the test, mice were individually put in the empty arena for habituation for 5 min. 24 h later, the mice were exposed to two identical objects within the arena for 5 min. On the test day, 24 h later, one of the familiar objects was replaced with a novel one, and mice were allowed to explore the arena again for 5 min, during which the time spent near the novel and the familiar object was measured. A preference index was calculated as follows: (time near novel object − time near familiar object)/(time near novel object + time near familiar object) (Dix and Aggleton, 1999).

#### Y-Maze Paradigm

The Y-maze paradigm was used to evaluate spontaneous exploration, responsiveness to novel environments and spatial memory function, as previously described (Baratz et al., 2010). This test relies on the preference of rodents to explore new environments rather than familiar ones. The apparatus consists of three-armed black Plexiglass maze with arms separated by 120°. Each arm was identical (8 × 30 × 15 cm); however, different spatial cues were placed in each arm (i.e., a triangle, a square, or a circle). The start arm was chosen randomly. In the first session of the test, the mouse was put in the start arm of the arena and allowed to explore another arm while the third one was blocked for 5 min. The mouse was then returned to its home cage for 2 min. Meanwhile, the arena was cleaned with 70% ethanol. In the second session of the test after an interval of 2 min, all arms were open for exploration for 2 min. The time the mouse spent in the familiar arm and in the new arm was measured. A preference index was calculated as follows: (time in new arm − time in familiar arm)/(time in new arm + time in familiar arm) (Dix and Aggleton, 1999).

### Immunohistochemical Staining

Immunohistochemistry studies were performed on mouse hippocampal (CA3 and dentate gyrus) and lateral cortical tissue sections obtained from animals euthanized on day 3 after injury; 72 h after injury and initiation of the various treatments, mice were anesthetized with a combination of ketamine (100 mg/kg) and xylazine (10 mg/kg) and perfused transcardially with 10 ml phosphate buffered saline (PBS) followed by 20 ml of 4% paraformaldehyde (PFA) in 0.1 M phosphate buffer, pH 7.4. Brains were removed, fixed overnight in 4% PFA, and then placed in 1% PFA. Prior to sectioning, brains were transferred to 30% sucrose for 48 h. Frozen coronal sections (30 μm) were cut on a cryostat and collected serially from bregma ∼−1.28 mm to ∼−2.4 mm. The sections were placed in a cryoprotectant solution containing phosphate buffer, ethylene glycol, and glycerin, and stored at −20°C. In all groups of mice, sections from both hemispheres were stained and analyzed. Random free-floating sections were blocked by 0.1% Triton X-100 in PBS (PBST) and 10% normal horse serum for 1 h at 25°C and incubated for 48 h at 4°C with appropriate primary antibodies specific to key features of interest in our studies. Controls consisted of omission of primary antibodies. Brain sections from multiple groups were reacted in the same well and evaluations were made by an observer blinded to the treatment groups.

#### FJB and NeuN Immuno-Staining

To assess the neurodegeneration occurring at an early stage following mTBI, we performed double-staining using Fluoro Jade B (FJB), a marker of degenerating neurons, and neuronal nuclear antigen (NeuN), a marker of mature neurons. Sections were incubated for 48 h with a mouse primary antibody that detects NeuN (Millipore; MAB377, 1:50 in incubation buffer). After NeuN incubation, sections were washed and incubated with a Cy3 labeled anti-mouse secondary antibody (Jackson; 715-165-150, 1:300 incubation buffer). The probed sections were mounted onto 2% gelatin coated slides and stained with FJB (Millipore; AG310), as described by Schmued and Hopkins (2000). The brain sections were observed using a Zeiss Axiovert 200 fluorescence microscope (Zeiss), using a ×20 magnification. For each brain, three to four sections were used to capture six to eight images from the hippocampus (CA3 and dentate gyrus) and cortex. A ratio of the number of degenerating neurons (FJB positive cells) to the number of mature neurons (NeuN positive cells) was used as an index of trauma-induced neuronal degeneration. Images were taken from both sides of the brain, and subsequently, were quantitively analyzed using ImageJ 1.50i software to provide a mean value for each brain region.

#### GFAP/Iba-1 Immuno-Staining

To assess changes in the expression of reactive astrocytes and activated microglia, sections were incubated for 48 h with rabbit glial fibrillary acidic protein (GFAP) primary antibody (Dako; Z0334, 1:500 in incubation buffer) or with rabbit ionized calcium-binding adapter molecule 1 (Iba-1) primary antibody (Wako; 019-19741, 1:500 in incubation buffer), respectively. The sections then were washed and incubated with donkey anti-rabbit secondary antibody (Abcam; Alexa Fluor^®^ 594 ab150064, 1:300 in incubation buffer). After rinsing with PBST, sections were mounted onto 2% gelatin-coated slides. The brain sections were observed using a Leica SP5 confocal microscope (Leica, Germany), using a ×40 magnification. For each brain, three to four sections were used to capture six to eight images from the hippocampus (CA3 and dentate gyrus) and cortex. Images were taken from both sides of the brain, and subsequently, were quantitively analyzed using the Imaris software (Bitplane AG, Zurich, Switzerland) to provide a mean value for each brain region.

### Western Blotting

To assess the phosphorylated PI3K/PKA levels in mouse cortex and hippocampus, brains were collected following cervical translocation 1 h to 1 week following mTBI induction. Right/left cortex and right/left hippocampus were separated and frozen in liquid nitrogen, then stored in −80°C. Thereafter, brains were dissociated and homogenized in a buffer lysis (Tissue Protein extraction Reagent, Pierce) supplemented with a protease inhibitor cocktail (Halt Protease Inhibitor Cocktail, Sigma–Aldrich) using a Teflon pestle homogenizer. Homogenates were centrifuged for 15 min at 4°C and 14,000 r/min and the supernatant liquids were separated from the precipitates and stored at −80°C. Sample buffer was added to the samples and then stored at −20°C. Samples were heated to 90°C for 3 min; 30 μl of each sample was then loaded and run on 4–20% Mini-Protean TGX gels (Bio-Rad; 456-1094) followed by transfer onto nitrocellulose membranes (Bio-Rad; 1704159) by a transfer system (Trans-Blot Turbo, Bio-Rad). Afterward, blots were blocked for 1 h at room temperature, with Tris-buffered saline, containing 0.01% Tween-20 and 5% BSA or powdered milk. Membranes were then incubated overnight at 4°C with a rabbit primary phospho-PI3K antibody (Cell signaling; 4228, 1:1000) or a rabbit primary phospho-PKA antibody (Cell signaling; 5661, 1:900) followed by washings with TTBS. Membranes were then incubated at room temperature for 1 h with horseradish peroxidase-conjugated goat anti-rabbit antibody (Jackson; 111-035-003, 1:10,000). Bands were then visualized using enhanced chemiluminescence reagents for 1 min (enhanced chemiluminescence assay) (Millipore, Billerica, MA, United States) by Viber Fusion FX7 imaging system (Viber Lourmat, France). A densitometry analysis of the detected signal was made using ImageJ software. Uniform loading was verified by stripping and re-probing with a mouse primary α-tubulin antibody for 30 min in room temperature (Santa Cruz; sc-53030, 1:10,000), followed with conjugated goat anti-mouse secondary antibody (Jackson, 115-035-003, 1:10,000). The value of each sample was determined by the ratio of p-PI3K or p-PKA and α-tubulin. Averages of control values in each membrane were set to 1, and all other samples were calculated accordingly.

### Data Analysis

All results are presented as mean ± SEM and were analyzed by SPSS V 25 software. One-way ANOVA tests were performed for comparisons between multiple datasets, followed by Fisher’s least significant difference (LSD) *post hoc* analysis, when found significant. Significant values between means are expressed as ^∗^*p* < 0.05, ^∗∗^*p* < 0.01, ^∗∗∗^*p* < 0.001.

## Results

### mTBI Exposure and Liraglutide/Twincretin Treatment Has No Effect on Anxiety and General Well-Being of the Mice

Following mTBI exposure and treatment with either liraglutide or twincretin, male ICR mice were evaluated using the elevated plus maze at 7 and 30 days post mTBI among separate cohorts. Specifically, two parameters were evaluated, the anxiety-like behavior and locomotor activity of the mice, by recording the time spent in the open arms of the maze and the total number of entrances to each arm of the maze, respectively.

**FIGURE 2:**
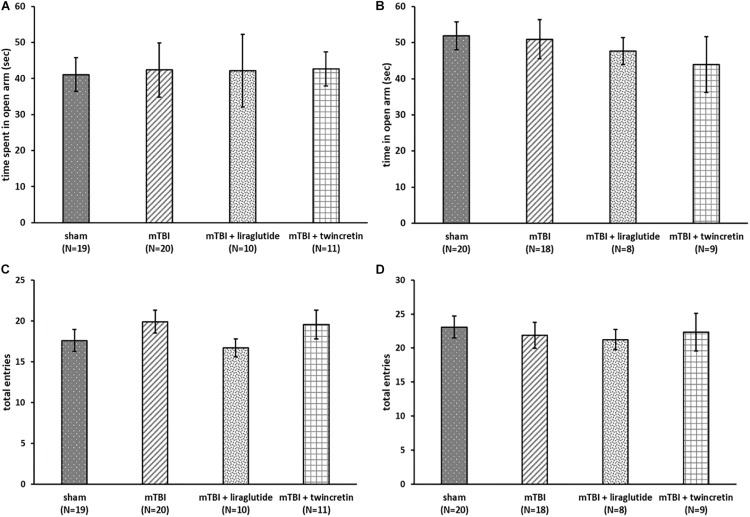
mTBI exposure and liraglutide/twincretin treatment do not impact the overall condition of the mice, as assessed in the elevated plus maze at both 7 and 30 days post trauma. Anxiety-like behavior assessment at 7 **(A)** and 30 **(B)** days post injury. The time spent in open arms of the maze was recorded and compared between groups. One-way ANOVA analysis showed that there were no differences between groups and that all groups of mice spent equivalent times in the open arms at both 7 and 30 days post mTBI [**A:** *F*(3,56) = 0.064; NS and **B:** *F*(3,51) = 0.374; NS]. Values are mean ± SEM. Locomotor activity at 7 **(C)** and 30 **(D)** days post injury. The number of total entrances to the arms of the maze was recorded and compared between groups. One-way ANOVA analysis demonstrated that there were no differences between the groups and all groups of mice had an approximately equal number of entrances to the arms at both 7 and 30 days post mTBI [**C:** *F*(3,56) = 0.830; NS, and **D:** *F*(3,51) = 0.218; NS]. Values are mean ± SEM.

As illustrated in Figures 2A,B, there were no differences in the time spent in the open arms between all groups at both time points tested, indicating that anxiety-like behavior of the mice was not affected either by the injury or by treatment with either liraglutide or twincretin.

Evaluation of the locomotor activity of the same animals revealed all groups had an approximately equal number of entrances to the arms of the maze and were not differentiated from one another at either 7 or 30 days post injury (Figures 2C,D), suggesting that the locomotor activity of the mice was unaffected by mTBI challenge or by liraglutide/twincretin treatment. Hence, mTBI and drug actions on anxiety and locomotor were not evident and cannot be considered caveats when evaluating actions on cognition.

### Liraglutide or Twincretin Treatment Reverses mTBI-Induced Cognitive Impairments in Mice

To assess the effects of liraglutide and twincretin treatment on post mTBI memory formation, the NOR and Y-maze paradigms were performed on mice at 7 and 30 days post injury, which received sham procedure, mTBI, or mTBI followed by either liraglutide or twincretin treatment. The time that mice spent investigating the novel/familiar object or arm was recorded, and a preference index was then calculated.

We have previously demonstrated that mice challenged with mTBI demonstrate cognitive impairments in visual and spatial memory, as evaluated by the NOR and Y-maze paradigms, respectively, and, notably, that liraglutide and twincretin ameliorate these impairments (Li et al., 2015; Tamargo et al., 2017). We show, herein, similar experiments with a direct comparison between the two drugs using doses relevant to clinical utilization of these agents [specifically, the selected liraglutide dose (246.7 μg/kg/day) translates across species to the human dose routinely used in T2DM; whereas the twincretin dose (50 μg/kg/day) translates to one-fifth of the dose used in humans]. There are two rationales for this approach. First is a cross validation with our previous work. Second, and particularly importantly, the immunohistochemical and molecular studies detailed below have not been previously undertaken for either liraglutide or twincretin, and were performed using brain tissue from a population of behaviorally evaluated animals.

**FIGURE 3:**
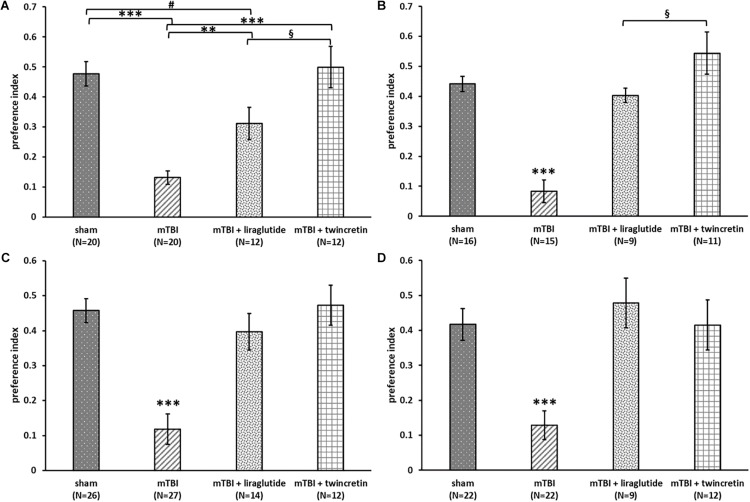
Liraglutide and twincretin treatments lead to improvement in mTBI-induced cognitive impairments at both 7 and 30 days post trauma. Visual memory was assessed using the novel object recognition (NOR) paradigm at 7 days **(A)** and 30 days **(B)** post injury. Preference index was used to represent the relative time that animals spend exploring a novel object compared to a familiar one, which reflects visual memory. One-way ANOVA test followed by Fisher’s LSD *post hoc* analysis revealed that the preference index of mTBI group was significantly lower than all other groups at both time points [**A:** *F*(3,60) = 17.135; *p* < 0.001 and **B:** *F*(3,47) = 23.22; *p* < 0.001, ^∗∗^*p* < 0.01, ^∗∗∗^*p* < 0.001). The preference index of the liraglutide treated group was significantly lower than sham and twincretin treated group at 7 days (*#p* < 0.05 vs. sham and §*p* < 0.05 vs. mTBI + liraglutide), while only lower than twincretin treated group at 30 days (§*p* < 0.05 vs. mTBI + liraglutide). Values are mean ± SEM. Spatial memory was evaluated using the Y-maze paradigm at 7 **(C)** and 30 days **(D)** post injury. Preference index was used to represent the relative time that animals spent exploring a novel arm of the maze compared to a familiar one, reflecting spatial memory. One-way ANOVA followed by Fisher’s LSD *post hoc* analysis revealed that the preference index of mTBI group was significantly lower than all other groups at both time points [**C:** *F*(3,75) = 15.851; *p* < 0.001 and **D:** *F*(3,61) = 9.811; *p* < 0.001, ^∗∗∗^*p* < 0.001]. Treatment with either liraglutide or twincretin led to similar normalization. Values are mean ± SEM.

The NOR test was used to examine visual recognition memory. mTBI-challenged mice suffered a significant visual memory deficit, evidenced by a reduced novel object exploration time compared to sham mice at both 7 and 30 days post injury (*p* < 0.001, Figures 3A,B). This deficit was substantially ameliorated by either liraglutide or twincretin treatment (*p* < 0.01). Importantly, at both 7 and 30 days post injury in separate cohorts of mice, liraglutide treatment demonstrated only partial recovery, as compared to controls, whereas twincretin resulted in full recovery. Notably, twincretin treated mTBI mice demonstrated significantly higher object discriminatory preference as compared to liraglutide treated mice (*p* < 0.05).

The Y-maze paradigm was implemented to evaluate the spatial memory. At both time points evaluated, mTBI-challenged mice experienced spatial memory impairment, as compared to sham mice (*p* < 0.001, Figures 3C,D); spending similar times exploring each arm of the maze. Treatment with either liraglutide or twincretin fully attenuated this impairment at both times tested (*p* < 0.001), with no observed superiority for one or the other treatments.

### Liraglutide or Twincretin Treatment Reduces mTBI-Induced Neuronal Degeneration

**FIGURE 4:**
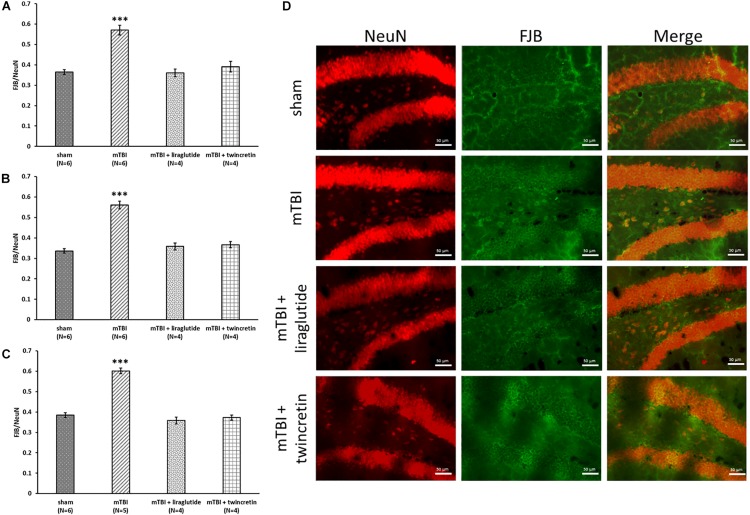
Liraglutide and twincretin treatments mitigate mTBI-induced neurodegeneration, 72 h post trauma. Graphs showing the quantification of degenerating neurons as the ratio of FJB/NeuN in the temporal cortex **(A)**, CA3 region **(B)**, and the dentate gyrus **(C)**. One-way ANOVA test followed by Fisher’s LSD *post hoc* analysis demonstrated that mTBI induction led to significant elevation in the ratio of degenerating neurons compared to sham tissues in all regions tested [**A:** *F*(3,16) = 26.695; *p* < 0.001, **B:** *F*(3,16) = 48.130; *p* < 0.001, and **C:** *F*(3,15) = 73.021; *p* < 0.001, ^∗∗∗^*p* < 0.001]. Treatment with either liraglutide or twincretin caused a significantly lower FJB/NeuN ratio compared to mTBI (^∗∗∗^*p* < 0.001), suggesting a reduction in the development of neurodegeneration following the injury. Values are mean ± SEM. **(D)** Representative images of immunohistochemical staining in the dentate gyrus are presented. NeuN positive cells are shown in red and FJB positive cells are shown in green; merged images show the overlap of FJB/NeuN positive cells. The scale bars are 50 μm.

Assessment of degenerating neurons induced by mTBI was undertaken immunohistochemically using FJB staining and a NeuN antibody at 72 h following the injury. Three different brain areas were evaluated: the temporal cortex, CA3 region, and the dentate gyrus. FJB is widely used to label neurons that are undergoing degenerative processes, since it specifically binds to degenerating neurons. NeuN antibody is routinely used to detect mature neurons, as it specifically recognizes the DNA-binding neuron-specific protein NeuN. A ratio of the degenerating neurons in each area was determined as: number of cells undergoing degeneration/number of mature cells (FJB/NeuN). The larger this ratio, the greater the extent of the neuronal degeneration. mTBI challenge resulted in a significant elevation in degenerating neurons as compared to the sham procedure (*p* < 0.001, Figure 4). This cellular loss was diffuse and occurred throughout each of the three evaluated brain regions. Importantly, mTBI-induced neurodegeneration was both substantially and significantly mitigated by treatment with either liraglutide or twincretin (*p* < 0.001), and the FJB/NeuN ratios from drug-treated groups were similar to those of controls. There was no additional therapeutic benefit observed for twincretin compared to liraglutide treatment.

### Liraglutide/Twincretin Treatment Mitigates mTBI-Induced Neuroinflammation by Reducing Activated Microglial Expression, but Does Not Impact Astrogliosis

**FIGURE 5:**
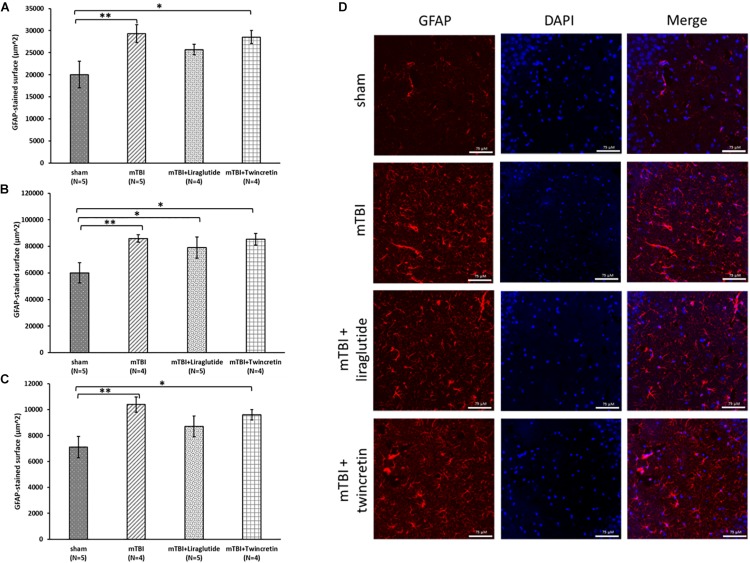
Liraglutide and twincretin treatments do not affect the mTBI-induced elevation in reactive astrocyte expression, 72 h post trauma. Graphs presenting quantification of the total surface labeled with GFAP in the temporal cortex **(A)**, CA3 region **(B)**, and the dentate gyrus **(C)**. One-way ANOVA followed by Fisher’s LSD *post hoc* analysis revealed that mTBI induction resulted in a significant elevation in GFAP expressing cells compared to sham tissues in all regions tested [**A:** *F*(3,14) = 3.917; *p* < 0.05, **B:** *F*(3,15) = 3.785; *p* < 0.05, and **C:** *F*(3,15) = 4.178; *p* < 0.05, ^∗∗^*p* < 0.01]. Treatment with neither liraglutide nor twincretin affected this elevation as compared to mTBI. Liraglutide treatment following mTBI did not prevent the elevation in GFAP expression as compared to sham mice in the CA3 region (^∗^*p* < 0.05); GFAP expression in the twincretin treatment group was significantly higher than control in all three regions (^∗^*p* < 0.05). Values are mean ± SEM. **(D)** Representative images of immunohistochemical staining in the CA3 region are presented. GFAP positive cells are shown in red and DAPI positive cells are shown in blue. The scale bars are 75 μm.

**FIGURE 6:**
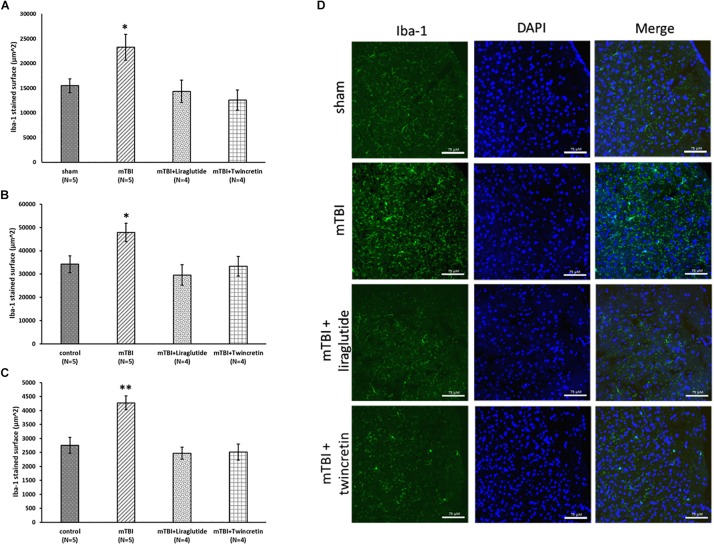
Liraglutide and twincretin treatments reduce the mTBI-induced elevation in activated microglia expression, 72 h post trauma. Graphs presenting quantification of the total surface labeled with Iba-1 in the temporal cortex **(A)**, CA3 region **(B)**, and the dentate gyrus **(C)**. One-way ANOVA followed by Fisher’s LSD *post hoc* analysis demonstrated that mTBI induction led to a significant elevation in Iba-1 expressing cells compared to sham tissues in all regions tested [**A:** *F*(3,14) = 5.117; *p* < 0.05, **B:** *F*(3,14) = 4.060; *p* < 0.05, and **C:** *F*(3,14) = 11.102; *p* < 0.01, ^∗^*p* < 0.05 in the cortex and CA3 and ^∗∗^*p* < 0.01 in the dentate gyrus]. Treatment with either liraglutide or twincretin following mTBI induction attenuated this elevation as compared to mTBI with no treatment. Values are mean ± SEM. **(D)** Representative images of immunohistochemical staining in the temporal cortex are presented. Iba-1 positive cells are shown in green and DAPI positive cells are shown in blue. The scale bars are 75 μm.

Evaluation of neuroinflammatory processes occurring subsequent to mTBI exposure was obtained by immunohistochemical staining using two different antibodies; GFAP labels reactive astrocytes and Iba-1 labels microglia. These were likewise studied across three different brain regions: temporal cortex, CA3 region, and the dentate gyrus, 72 h post injury. Increases in the expression and activation of astrocytes and microglia, together with acute upregulation of proinflammatory cytokines such as interleukin (IL)-1β, tumor necrosis factor (TNF)-α, and IL-6 (Patterson and Holahan, 2012), are key components in injury processes evident following mTBI. mTBI exposure induced a significant elevation in astrocyte and microglial reactivity within all three brain regions examined (*p* < 0.01 and *p* < 0.05, respectively, Figures 5, 6). Treatment with neither liraglutide nor twincretin affected the mTBI-induced elevation in GFAP expression (Figure 5). However, both treatments equally reduced Iba-1 elevated immunoreactivity caused by mTBI challenge (*p* < 0.05, Figure 6).

### mTBI Induction Causes a Reduction in PKA Expression in the Ipsilateral Cortex but Does Not Affect PI3K Levels

**FIGURE 7:**
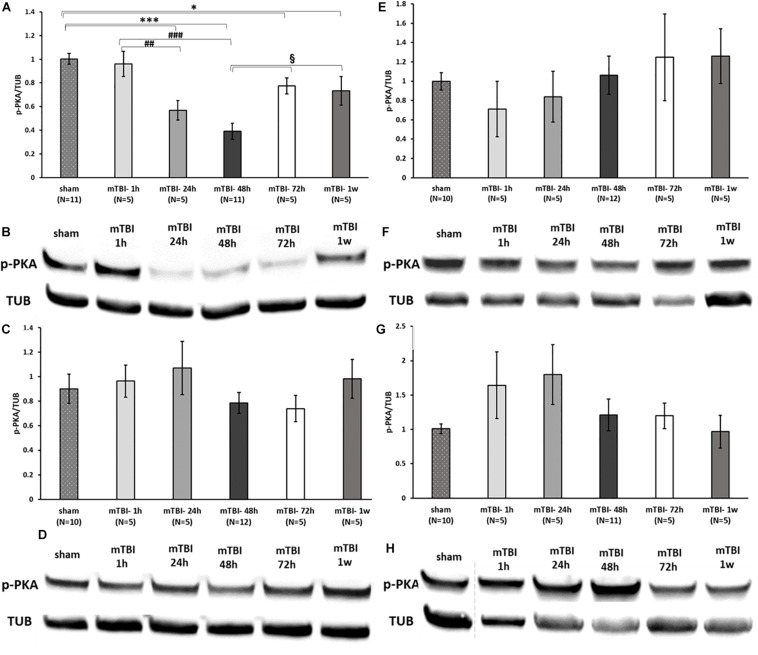
mTBI results in a decline in p-PKA levels in ipsilateral cortex. Graphs presenting p-PKA expression in ipsilateral/right **(A)** and contralateral/left **(C)** cortex and ipsilateral **(E)** and contralateral **(G)** hippocampus in sham and 1 h to 1 week following mTBI. One-way ANOVA followed by Fisher’s LSD *post hoc* analysis revealed a significant reduction in p-PKA expression in cortex right 24 h to 1 week following mTBI [**A:** *F*(5,36) = 12.060; *p* < 0.001, ^∗∗∗^*p* < 0.001 sham vs. 24, 48 h and ^∗^*p* < 0.05 sham vs. 72 h, 1 w], ^##^*p* < 0.01, ^###^*p* < 0.001 mTBI 1 h vs. mTBI 24 h and vs. 48 h, respectively, when the maximal reduction present in 48 h was followed by an elevation in the levels of PKA (72 h–1 week, §p < 0.05 vs. 48 h). Blots from all other regions remained unchanged by the injury [**C:** *F*(5,36) = 0.809; NS, **E:** *F*(5,36) = 0.585; NS, **G:** *F*(5,35) = 1.373; NS]. Values are mean ± SEM. **(B,D,F,H)** Representative images of gel electrophoresis followed by immunoblot analysis using antibodies against p-PKA and α-tubulin in ipsilateral cortex, contralateral cortex, ipsilateral hippocampus, and contralateral hippocampus, respectively. A crop of the original image was performed in 7F at loading site number 2 of the gel due to an abnormal value of a control sample that was therefore excluded from the statistical analysis.

**FIGURE 8:**
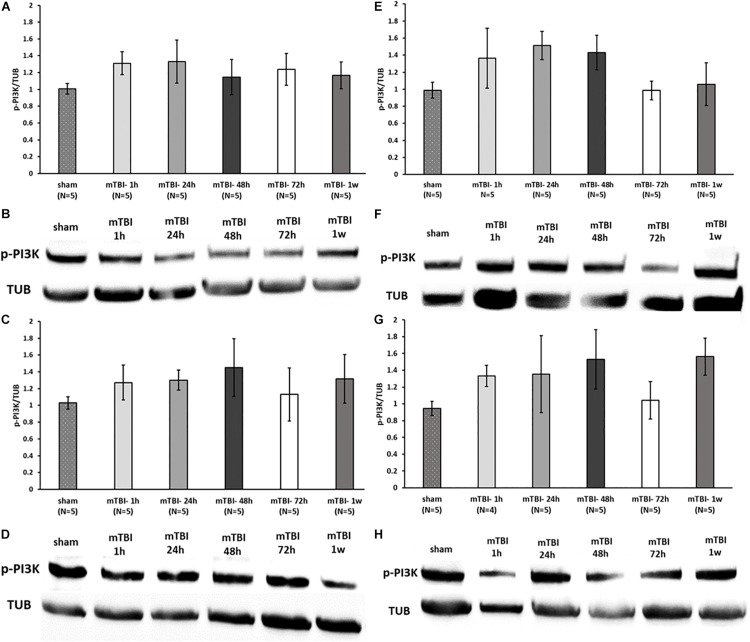
mTBI challenge does not affect the p-PI3K levels in the cortex and hippocampus. Graphs showing p-PI3K expression in ipsilateral/right **(A)** and contralateral/left **(C)** cortex and ipsilateral **(E)** and contralateral **(G)** hippocampus in sham and 1 h to 1 week following mTBI. One-way ANOVA analysis showed no significant differences between the groups in all brain regions, indicating that mTBI induction does not affect the p-PI3K levels at the times tested [**A:** *F*(5,24) = 0.538; NS, **C:** *F*(5,24) = 0.365; NS, **E:** *F*(5,24) = 1.236; NS, **G:** *F*(5,23) = 0.813; NS]. Values are mean ± SEM. **(B,D,F,H)** Representative images of gel electrophoresis followed by immunoblot analysis using antibodies against p-PI3K and α-tubulin in ipsilateral cortex, contralateral cortex, ipsilateral hippocampus, and contralateral hippocampus, respectively.

To assess the effect of mTBI on the levels of neuroprotective proteins downstream to activation of GLP-1/GIP receptors, Western blot analysis was performed to quantify the levels of the phosphorylated form of PKA and PI3K in a time-dependent manner (1, 24, 48, and 72 h and 1 week post trauma) in four different brain regions: ipsilateral (right)/contralateral (left) cortex, and ipsilateral/contralateral hippocampus. Following activation of GLP-1R/GIPR, activated PKA is involved in the cAMP/PKA/CREB signaling pathway, whereas activated PI3K is involved in the PI3K/AKT pathway (Li et al., 2010b; Kim et al., 2017; Athauda and Foltynie, 2018). Both pathways promote cell survival as well as neurite outgrowth, memory formation, and inhibition of apoptosis. mTBI challenge resulted in reduced levels of p-PKA in the ipsilateral cortex evident between 24 h to 1 week post mTBI (24–48 h: *p* < 0.001, 72 h–1 week: *p* < 0.05), whereas all other brain regions remained unaffected [Figure 7 (please note that in Figure 7F, sample no. 2 of the original membrane was cropped from the image, as noted in the figure legend)]. In contrast, mTBI did not significantly alter PI3K phosphorylation levels across any of the brain regions (Figure 8).

### Liraglutide/Twincretin Treatment Attenuates mTBI-Induced Decline in p-PKA Expression in the Ipsilateral Cortex

**FIGURE 9:**
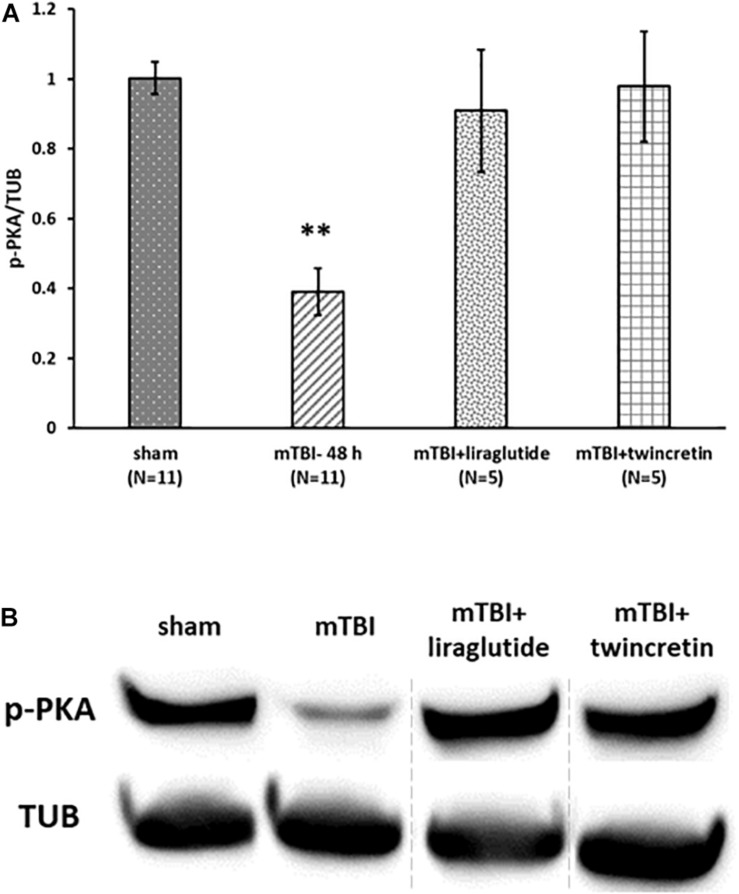
Liraglutide and twincretin treatments block mTBI-induced reduction in p-PKA expression in the ipsilateral cortex. **(A)** Graph showing the p-PKA expression levels in ipsilateral/right cortex 48 h following mTBI. One-way ANOVA followed by Fisher’s LSD *post hoc* analysis revealed a significant reduction in p-PKA expression in ipsilateral injured cortex 48 h following mTBI as compared to sham [*F*(5,28) = 12.603; *p* < 0.001, ^∗∗^p < 0.01]. Treatment with either liraglutide or twincretin reduced the decline observed in mTBI affected brains (^∗∗^*p* < 0.01, respectively). Values are mean ± SEM. **(B)** Representative images of gel electrophoresis followed by immunoblot analysis using antibodies against p-PKA and α-tubulin in the ipsilateral cortex. A crop of the original image was performed, and sample number 3 was replaced by sample number 8, since the sample of mTBI + liraglutide was on loading site number 8 of the gel.

As mTBI induced a significant change in expression of only p-PKA in ipsilateral cortex (Figure 7A), the effect of liraglutide or twincretin on p-PKA in this area was assessed at the most affected time point: 48 h. Treatment with either liraglutide or twincretin rescued the significant reduction in p-PKA expression 48 h following mTBI induction in the ipsilateral cortex (*p* < 0.01, Figure 9, please note that the order of the samples is not as in the original image of the membrane). Both treatments had similar efficacy.

## Discussion

The present study proposes a potential therapeutic strategy to treat mTBI sequalae. Incretin analogs have successfully been used to treat T2DM for many years and are well tolerated. In addition to their insulinotropic actions, incretins promote pancreatic β-cell proliferation and inhibit apoptotic processes in these cells (Baggio and Drucker, 2007; Seino et al., 2010). These protective and trophic effects, along with enhanced memory formation, are evident in the brain (Perry et al., 2002; During et al., 2003; Nyberg et al., 2005; Li et al., 2009, 2010b; Paratore et al., 2011) since incretins cross the blood–brain barrier (BBB) and their receptors are expressed on neurons throughout the brain (Hamilton and Holscher, 2009; Figueiredo et al., 2011; Spielman et al., 2017). Numerous preclinical studies have emphasized the neuroprotective and neurotrophic actions of incretins and analogs in different rodent models of neurological disorders and neurodegenerative diseases, in which incretin mimetics enhanced memory and learning, promoted neuronal survival, induced neurogenesis, and inhibited neuroinflammation (Perry et al., 2007; Bertilsson et al., 2008; Li et al., 2009, 2016; Holscher and Li, 2010; McClean et al., 2011; Faivre and Holscher, 2013; Kim et al., 2017). Since findings from these preclinical studies were promising, GLP-1 analogs are now being investigated in humans in clinical trials for treatment of Alzheimer’s disease (Egefjord et al., 2012; Gejl et al., 2016) and Parkinson’s disease (Aviles-Olmos et al., 2013; Athauda et al., 2017, 2019). While several pharmacological treatments besides incretin analogs have been identified for these neurodegenerative diseases, TBI lacks any evidence-based treatment (Levin and Diaz-Arrastia, 2015). Therefore, investigation for a potential therapy that may alleviate the cognitive, behavioral, and emotional deficits occurring after TBI is important.

The pathophysiology of mTBI is complex and characterized especially by neuronal cell death and axonal damage induced primarily by the direct physical impact itself and secondarily by oxidative stress, neuroinflammation, mitochondrial dysfunction, DNA damage, and other pathological processes (Maas et al., 2008; Choe, 2016; McInnes et al., 2017). Since incretins have the capability to intervene in more than one of these pathological events (Perry et al., 2002; Nyberg et al., 2005; Iwai et al., 2006; Li et al., 2010b; Paratore et al., 2011; Yu et al., 2016; Verma et al., 2017), incretin mimetics have the potential to serve as neuroprotective agents following mTBI.

Our previous studies focused on the effects of several clinically relevant incretin analogs on mTBI-induced impairments in cellular and rodent mTBI models following administration of clinically translatable doses (Glotfelty et al., 2019). Specifically, we demonstrated the efficacy of exendin-4 (a GLP-1 analog), liraglutide (a long acting GLP-1 analog), twincretin (a dual GLP-1/GIP analog), and PT302 (a sustained release formulation of the GLP-1 analog Exanatide) to mitigate cellular damage associated with mTBI pathologies such as oxidative stress, glutamate induced toxicity, and apoptotic cell death. Moreover, these agents were successful in ameliorating the cognitive deficits evident following exposure to mTBI in mice (Rachmany et al., 2013; Li et al., 2015; Tamargo et al., 2017; Bader et al., 2019; Glotfelty et al., 2019) and other mTBI-induced brain pathologies such as neurodegeneration and neuroinflammation (Bader et al., 2019).

In the current study, we aimed for a more comprehensive and comparative evaluation of the potential benefit of enhancing incretin activity in the brain on mTBI-induced cognitive, cellular, and molecular impairments. To this end, we examined whether liraglutide and twincretin could be used as neuroprotective agents in a preclinical mTBI mouse model. In addition, we sought to assess whether there was a therapeutic advantage in jointly activating the two incretin receptors by twincretin vs. single GLP-1R activation by liraglutide. Liraglutide is an FDA approved treatment for T2DM, whereas twincretin is under investigation in ongoing clinical trials to treat T2DM (Finan et al., 2013; Frias et al., 2017; Portron et al., 2017; Schmitt et al., 2017).

Mice were exposed to mTBI using our well-characterized closed-head weight drop model that mimics key mTBI symptoms and pathologies in humans (Zohar et al., 2003; Milman et al., 2005), and post injury were administered either liraglutide at a translatable dose to humans [247.6 μg/kg: equivalent to a 1.8 mg dose in a human of approximately 88.8 kg weight (the mean weight of a male American (U.S. Department of Health and Human Services et al., 2016)], following normalization to body surface area across species in line with FDA guideline [U.S. Department of Health and Human Services Food and Drug Administration 2005 (U.S. Department of Health and Human Services et al., 2005)] or twincretin in a previously used efficacious dose in rodent models (50 μg/kg; Finan et al., 2013; Tamargo et al., 2017) by s.c. injections once a day for 7 consecutive days. Subsequently, mice were assessed behaviorally and cognitively, and brains of different mice cohorts were used for immunohistochemical staining and Western blot evaluations.

Mild TBI impairment in cognitive abilities was manifested in the NOR and Y-maze paradigms at both 7 and 30 days post mTBI (evaluated in separate cohorts of mice for each time point). Importantly, treatment with either liraglutide or twincretin protected against these short and longer term cognitive impairments, without impacting measures of anxiety or locomotor activity. Reports in the literature indicate that some TBI models can induce anxiety-like behavior (Washington et al., 2012), and there are accounts of anti-anxiety and antidepressant effects among GLP-1 and its analogs (Isacson et al., 2011; Anderberg et al., 2016). We avoided anxiety symptoms following the injury by using short-term anesthesia at the time of the TBI impact induction, to ensure that animals were unaware of the procedure. Hence, untoward actions on either anxiety or motor activity cannot be considered confounds when evaluating liraglutide and twincretin mitigation of mTBI-induced cognitive impairments, and these positive actions are in line with prior reports regarding the cognitive alleviation provided by incretin analogs following an mTBI challenge (Rachmany et al., 2013; Li et al., 2015; Tamargo et al., 2017; Bader et al., 2019; Glotfelty et al., 2019), as well as in Alzheimer’s disease models (Faivre and Holscher, 2013; Shi et al., 2017; Batista et al., 2018; Glotfelty et al., 2019).

In the NOR paradigm, treatment with twincretin, notably, resulted in significantly greater improvement in visual memory over that achieved by liraglutide, which likely is due to activation of both GLP-1R and GIPR, in contrast to liraglutide activation of GLP-1R alone. Several studies comparing the efficacy of dual GLP-1R/GIPR vs. a GLP-1R agonist across various pathologies, likewise, demonstrated a therapeutic advantage for the dual agonist. This was observed in diabetic (Finan et al., 2013), Parkinson’s disease (Yuan et al., 2017), and ischemia (Han et al., 2016) models. In these prior studies, the same doses of the two substances were evaluated. In our study, however, the twincretin dose appraised (50 μg/kg) was, of note, some fivefold lower than liraglutide (247.6 μg/kg). Similarly, in the Y-maze paradigm in which an equal mitigation of mTBI-induced spatial memory loss was achieved with the two incretin mimetics, twincretin achieved this at one-fifth of the dose of liraglutide.

Our preliminary, dose-finding studies with twincretin in cellular and animal models demonstrated its greater potency over a single GLP-1R agonist at equimolar doses, and supported the evaluation of the selected twincretin 50 μg/kg dose (as opposed to the 247.6 μg/kg dose), as the former appeared to be at the top end of the linear portion of the dose–response curve. In this context, twincretin provides a potential advantage in terms of potency over liraglutide, as in ongoing clinical trials twincretin (also known as RG7697 and NNC 0090-2746; Frias et al., 2017; Portron et al., 2017; Schmitt et al., 2017) is currently being evaluated at a dose of 1.8 mg, and appears to be well tolerated and efficacious. As noted, the clinically approved dose of liraglutide is also 1.8 mg in T2DM (i.e., 247.6 μg/kg in a mouse). Hence, in synopsis, a fivefold lower dose of twincretin provided similar or greater activity across outcome measures, as compared to a clinically translatable dose of liraglutide.

A caveat of our study is that we were unable to evaluate twincretin in comparison to a clinically translatable dose of a single GIPR agonist. Whereas single GIPR agonists are available as pharmacological tools and several are long-acting (Glotfelty et al., 2019), none have translated into humans and hence no information is available as to a clinically translatable dose to support a comparative evaluation. Our prior studies evaluating a constant infusion of GIP in moderate TBI to overcome its rapid disappearance, demonstrated its ability to mitigate impairments when plasma GIP levels were raised by 2.2-fold (Yu et al., 2016). This prior study suggests that a relatively modest elevation in the level of an incretin can provide a biologically relevant effect, which is important in the light of the relatively small but quantifiable amount that enters the brain following systemic administration of incretin mimetic: with CSF levels achieving some 2% of plasma levels (Bader et al., 2019).

To gain a more thorough understanding of the causes of the cognitive impairments observed following mTBI, we examined select cellular changes in cortex and hippocampal regions associated with brain injury by performing immunohistochemical staining at 72 h post mTBI induction. This time point was chosen based on previous studies demonstrating that a number of biochemical changes are both evident and plateau at 72 h following mTBI (Tashlykov et al., 2007; Israelsson et al., 2009; Rubovitch et al., 2010). A significant elevation in neurodegenerative markers was observed across all regions evaluated. Treatment with each of the incretin analogs fully mitigated this. These results are in accord with elevations in neurodegenerative processes that occur following blast-mTBI and their amelioration by a clinically translatable dose of the GLP-1R agonist exendin-4 (Tweedie et al., 2016b). Moreover, a sustained-release formulation of exenatide (PT302) similarly prevented the elevation in neurodegenerative processes and prolonged neuronal loss following injury exposure in the same mTBI-model as in the present study (Bader et al., 2019).

We previously reported that our mTBI model, in line with other studies, induces key neuroinflammatory changes, including elevations in astrocyte and microglial expression, pro-inflammatory cytokine TNF-α levels, and expression of genes involved in inflammatory processes in several regions of the brain (Baratz et al., 2015; Tweedie et al., 2016a; Bader et al., 2019). In the current study, treatment with either liraglutide or twincretin reduced elevated microglial reactivity but failed to mitigate astrocyte changes, which is interesting in the light of recent studies suggesting that neurodegenerative processes triggered by neuroinflammation and the activation of microglia are mediated via A1 activated astrocytes (Liddelow et al., 2017; Yun et al., 2018). An increasing number of studies suggest that GLP-1R activation provides anti-inflammatory action, which our study supports. When evaluating these parameters at later time points, other studies have reported that incretin mimetics reduced elevated astrocyte expression in addition to microglial expression after 7–14 days of treatment (Yu et al., 2016; Barreto-Vianna et al., 2017; Yuan et al., 2017). One explanation for this difference, vs. our study, could be that the effect of these drugs on astrocytes occurs at a delayed time point compared to their effect on microglia, which was observed at 72 h following treatment initiation herein. Moreover, the GLP1R is highly expressed on microglia, in comparison to its expression on neurons and astrocytes (Iwai et al., 2006; Yun et al., 2018). Microglia have also been reported to generate and secrete GLP-1 (Kim et al., 2009; Kappe et al., 2012), and prior studies have demonstrated that GLP-1R agonists inhibit the activation of microglia in cellular studies following LPS stimulation and *in vivo* in response to numerous types of injury (Kim et al., 2009, 2017; Li et al., 2012; Gullo et al., 2017; Athauda and Foltynie, 2018; Wu et al., 2018; Yun et al., 2018). This results in a decline in the production of proinflammatory cytokines such as TNF-α and, as found in our study, a reduction in Iba-1 staining [as Iba-1 is a pan-microglial marker whose expression increases with microglial activation (Hopperton et al., 2018)].

Finally, to understand how our mTBI model and incretin analogs regulate neuroprotective protein expression, we examined the levels of PKA and PI3K phosphorylation in the cortex and hippocampus by Western blot. Contrary to the cellular pathways that incretins activate in the pancreas, those activated in brain remain to be fully characterized (Glotfelty et al., 2019). Multiple studies have proposed signaling pathways downstream to GLP-1R or GIPR activation in neurons (Perry and Greig, 2003; Li et al., 2010b; Holscher, 2014; McGrath et al., 2015; Kim et al., 2017; Athauda and Foltynie, 2018; Verma et al., 2018). In general, stimulation of either incretin receptors leads to the activation of the cAMP/PKA/CREB pathway that induces neuroprotection, inhibits apoptosis, regulates expression of genes that promote cell survival, and enhances memory formation (Glotfelty et al., 2019). This pathway also appears involved in GLP-1R mediated anti-inflammation (Wu et al., 2018). Agonist binding to the incretin receptors also activates the PI3K protein, which triggers several intracellular pathways involving the PKC, AKT/PKB, and MAPK proteins that ultimately promote cell proliferation and regulate expression of genes interfering with apoptotic processes (Perry and Greig, 2003; Holscher, 2014; McGrath et al., 2015; Kim et al., 2017; Athauda and Foltynie, 2018; Verma et al., 2018).

In our model, mTBI induction did not affect p-PI3K levels. Previous studies investigating the effects of mTBI on proteins involved in the PI3K/AKT signaling pathway demonstrated inconsistent findings; some reports showed a decline in the activation of the PI3K/AKT pathway following mTBI (Chen et al., 2012; Shen et al., 2017); others showed an upregulation of this pathway (Rubovitch et al., 2010; Wang et al., 2017) and still others showed non-significant effects (Gao et al., 2016). Differences in the activation of this pathway following mTBI are apparently model-, time-, and region-dependent. However, p-PKA levels were decreased following mTBI exposure, which is consistent with previous findings showing downregulation in the cAMP/PKA/CREB pathway following mTBI (Atkins et al., 2009; Wang et al., 2018). Finally, treatment with each of liraglutide and twincretin resulted in attenuation in the mTBI-induced reduced expression of p-PKA. This likely contributes to the manifested neuroprotective effects of liraglutide and twincretin. Our previous work on neuronal cells *in vitro* demonstrated that incretin analogs activate adenylyl cyclase, increase production of cAMP, and also activate CREB (Li et al., 2010a, b, 2015; Tamargo et al., 2017; Glotfelty et al., 2019), with twincretin having a particularly potent action (Tamargo et al., 2017).

## Conclusion

Liraglutide and twincretin effectively prevented cognitive impairments and secondary damages induced by mTBI in mice. Although these two incretin mimetics similarly and fully inhibited these deficits in the majority of our evaluations, there appeared to be a potency advantage for twincretin, as similar or greater actions were achieved at a fivefold lower dose than liraglutide (50 and 247.6 μg/kg, respectively). By contrast, in humans, both drugs are administered at the same dose (1.8 mg) (Frias et al., 2017). Future studies within our collaborative group are focused to investigate the neuroprotective effects of liraglutide, twincretin and related analogs across a range of doses in order to obtain a dose–response analysis to allow a more comprehensive comparison between incretin mimetics and inform the optimal conditions for a human mTBI clinical trial. The consistent efficacy of clinically translatable doses of incretin mimetics mitigating neurodegenerative, neuroinflammatory, and behavioral outcome measures across preclinical TBI models (Glotfelty et al., 2019) is reinforced by the current study, and provides yet further support for the selection of an incretin mimetic to evaluate this treatment strategy in humans.

## Data Availability Statement

The datasets generated for this study are included in the article/Supplementary Material. Requests to access the datasets should be directed to MB, miaad.bader@gmail.com.

## Ethics Statement

The animal study was reviewed and approved by the Ethics Committee of the Sackler Faculty of Medicine protocols M-14-050 and M-15-011.

## Author Contributions

CP, MB, BH, RD, and NG conceived the study. MB, YL, DT, NS, AB, and VR performed the experiments. RD contributed essential materials. MB, LT-y-R, VR, YL, and DT analyzed the data. MB, BH, LT-y-R, and NG wrote the manuscript. MB and NG responded to reviewers’ comments and revised the manuscript. All authors read and approved the final version of the manuscript.

## Conflict of Interest

RD is a co-inventor on patent applications (US2011/0166062 A1; US 12/999,285; “GIP-based mixed agonists for treatment of metabolic disorders and obesity”) owned by Indiana University that pertain to the twincretin peptide in this paper. The remaining authors declare that the research was conducted in the absence of any commercial or financial relationships that could be construed as a potential conflict of interest.
